# Barriers to accessing preventive health care among African-born individuals in King County, Washington: A qualitative study involving key informants

**DOI:** 10.1371/journal.pone.0250800

**Published:** 2021-05-10

**Authors:** D. Allen Roberts, Seifu Abera, Guiomar Basualdo, Roxanne P. Kerani, Farah Mohamed, Rahel Schwartz, Beyene Gebreselassie, Ahmed Ali, Rena Patel

**Affiliations:** 1 Department of Epidemiology, University of Washington, Seattle, Washington, United States of America; 2 Department of Medicine, University of Washington, Seattle, Washington, United States of America; 3 College of Arts and Sciences, University of Washington, Seattle, Washington, United States of America; 4 HIV/STD Program, Public Health – Seattle and King County, Seattle, Washington, United States of America; 5 Somali Health Board, Seattle, Washington, United States of America; 6 Ethiopian Health Council, Ethiopian Community in Seattle, Seattle, Washington, United States of America; 7 Eritrean Health Board, Seattle, Washington, United States of America; University of Calgary, CANADA

## Abstract

Studies of African immigrant health in the U.S. have traditionally focused on infectious diseases. However, the rising burden of non-communicable diseases (NCDs) indicates the increasing importance of general preventive health care. As part of a series of community health events designed for African-born individuals in King County, Washington, we administered key informant interviews (KIIs) with 16 health event participants, medical professionals, and community leaders to identify barriers and facilitators to use of preventive health care among African-born individuals. We used descriptive thematic analysis to organize barriers according to the socio-ecological model. Within the individual domain, KII participants identified lack of knowledge and awareness of preventive health benefits as barriers to engagement in care. Within the interpersonal domain, language and cultural differences frequently complicated relationships with health care providers. Within the societal and policy domains, healthcare costs, lack of insurance, and structural racism were also reported as major barriers. Participants identified community outreach with culturally competent and respectful providers as key elements of interventions to improve uptake. In conclusion, African immigrant communities face several barriers, ranging from individual to policy levels, to accessing health services, resulting in substantial unmet need for chronic disease prevention and treatment. Community-centered and -led care may help facilitate uptake and engagement in care.

## Introduction

The number of African-born immigrants in the U.S. has risen dramatically, with a fivefold increase since 1980 [1]. King County, Washington state (WA), has a larger percentage of foreign-born residents (24%) compared to the U.S. as a whole (14%), and the over 40,000 African-born residents make up about 8% of the foreign-born population in King County [2, 3]. The Seattle metropolitan statistical area, which contains King County, includes large Somali, Ethiopian, and Eritrean communities.

Immigrants face numerous challenges to obtaining effective health care, including issues surrounding language, legal status, stigma, reduced access to health insurance, and socioeconomic status [4, 5]. Studies of health needs of African immigrants in the U.S. have traditionally focused on infectious diseases [6]. However, due to the rising burden of non-communicable diseases (NCDs) and as more immigrants become long-term residents in the U.S., the prevention and management of chronic health conditions has become increasingly important [7]. Recent research has revealed high prevalence but low awareness of cardiometabolic disease among African immigrants in the U.S. [8, 9]. Other studies of African immigrants living in high-income settings have found low levels of health insurance [10] and linkage to primary care [11, 12] as well as low or delayed uptake of vaccination [13, 14], cancer screening [15, 16], antenatal care [17], and diabetes screening [18]. Therefore, identifying and addressing specific barriers to engagement in preventive health care (defined here as proactive measures taken to prevent or mitigate future illness) is needed to improve health outcomes of African immigrant populations in the U.S.

Prior qualitative research among African immigrants has primarily focused on barriers to accessing treatment services [19, 20]. Cultural attitudes and religious beliefs surrounding disease etiology and treatment efficacy may delay presentation to care [21–23]. Stigma has been consistently identified as a barrier to HIV testing and treatment [24–27] as well as to uptake of mental health services [28, 29]. Linguistic differences [5, 30, 31], legal status [32], cost [21], and provider discrimination [21, 22] may also impede effective care. Nevertheless, several evidence gaps remain. Few studies have specifically focused on barriers to preventive services, which may differ from those for seeking treatment. Furthermore, while many studies have reported barriers, few have explicitly solicited potential facilitators of increased engagement in preventive care [19]. Finally, African immigrants represent a heterogenous population with diverse experiences and beliefs, and barriers may differ depending on countries of origin, emigration destination, and local availability of services.

In light of limited data on preventive health behavior, our study objectives were to describe barriers to and potential solutions for increasing engagement in preventive health care among African immigrant communities in King County, WA. Key informant interviews (KIIs) offer a pragmatic approach to obtain insight into the nature of health issues and to plan health service delivery [33]. As part of an academic-community partnership, we participated in a series of 10 health promotion events geared for African-born individuals held at community locations in the greater Seattle area. During these events, we conducted a basic qualitative description study involving key informant interviews with African-born health event participants, medical professionals, and community leaders regarding access to and use of preventive services.

## Methods

### Study setting

This academic-community partnership was initially created to implement six health fairs held at apartment complexes housing high densities of African-born individuals in the greater Seattle area during April and May 2018, which has been described previously [27]. Notably, 33% of African-born participants did not have health insurance, 70% were overweight or obese, 30% had high blood pressure (>140 systolic or >90 diastolic), and 38% had elevated cholesterol. We additionally participated in four separate follow-up events hosted by community organizations between July and December 2018. The follow-up events included: a free community clinic hosted in an apartment complex housing many African-born individuals; an apartment complex community event which included health screenings; an annual, community health fair hosted by an ethnic health board; and a monthly social gathering and health fair hosted by another ethnic health council.

### Data collection

We conducted a basic qualitative description study [34] using key informant interviews with health event participants, African-born health professionals, and community leaders as described in detail previously [27]. These interviews covered barriers and facilitators to HIV testing (reported in [27]) as well as barriers and facilitators to preventive health care, which are reported here. Individuals eligible to be interviewed included African-born health fair participants, African-born medical professionals, or community leaders working with African-born groups. Event participants were purposively sampled during the ten total health events across gender, occupration, and birth place, while community leaders and medical professionals were identified via snowball sampling initiated by community partners assisting in organizing the health events. The interviews were completed by study staff (matching the interviewer with a native-speaking interviewee when possible) either in person or over the phone, with one team member (SA, male research coordinator and physician) conducting the majority. All invited interviewees agreed to participate, and each respondent was read an informed consent script, which included the specifc study objectives, and provided verbal consent. Interviews were conducted primarily in English (but occasionally in Amharic or Spanish) and took place in private locations at event sites or community centers. Each interview lasted about 30–40 minutes and included questions concerning barriers and facilitators for accessing preventive health care; community, cultural and gender norms; and preferred locations and providers (S1 File). For participants unfamiliar with the term “preventive care,” we provided examples such as annual health checkups, screenings for cancers or chronic diseases, and counseling for diet and exercise. The interview guide was designed to be open-ended and was not structured around a specific theoretical framework. While the same guide was used for all respondents, health event-specific questions were not asked to non-health event participants, though at times the questions were posed as hypotheticals. Interviews were not recorded (interviewers took field notes during the interview and updated any notes afer the interview as needed), participants were each interviewed only once, and field notes were not reviewed by participants. Each interviewed participant was asked to recommend 1–2 additional people who might be interested in interviewing for the study. After each interview, the interviewer summarized the key points from that interview. When the interviewer and study investigators felt that no new and dramatically different insights were being gained from additional interviews, we concluded that a saturation of major themes was reached and ceased to conduct further interviews [35, 36].

### Data analysis

We included two interviews with respondents who were born outside of Africa but employed as event site managers and had extensive experience organizing community events for African-born individuals. For this analysis, we excluded one interview that concluded prematurely and did not contain sufficient information for the focus on this analysis and one interview conducted with a study co-author who later contributed to data analysis (SA). Another KII participant was instrumental in event coordination and subsequently contributed to interpretation of the results (BG). That individual (BG) is included as a co-author on this manuscript. As BG did not contribute to the qualitative coding, thematic analysis, or selection of illustrative quotes, we decided to include his interview.

The field note transcripts obtained from the qualitative interviews were coded by one study member (GB) and reviewed by another (RP), with discrepancies resolved by consensus. Coding was conducted using Microsoft Word (Microsoft, Redmond, WA). We used descriptive thematic analysis with inductive coding [35]. The codes were arranged into thematic domains and subthemes in conjunction with illustrative quotations. Once we had greater clarity of the emerging themes, we decided to use the socio-ecological model [37] as a tool for organizing our thematic domains.

### Ethics statement

Ethical approval for study and oral informed consent procedures was obtained from the University of Washington Human Subjects Division.

## Results

### Key informant interviews

In total, we included 16 KIIs in this analysis. Respondent age ranged from 18 to 60 years, with over half of participants between 30 and 50 years old (Table 1). The majority of respondents were female (n = 11) and born in Ethiopia (n = 10). Eleven interviews were conducted with medical professionals or community leaders and five were conducted with event participants.

**Table 1:** Demographics and occupation of 16 key informant interview participants.

		N	(%)
Age	< 30	3	(19%)
	30–49	9	(56%)
	50+	4	(25%)
Gender	Male	5	(31%)
	Female	11	(69%)
Country of birth	Ethiopia	10	(63%)
	Other (Africa)	4	(25%)
	USA or Mexico	2	(13%)
Occupation	Medical professional	6	(38%)
	Community leader	5	(31%)
	Event participant	5	(31%)

**Fig 1:**
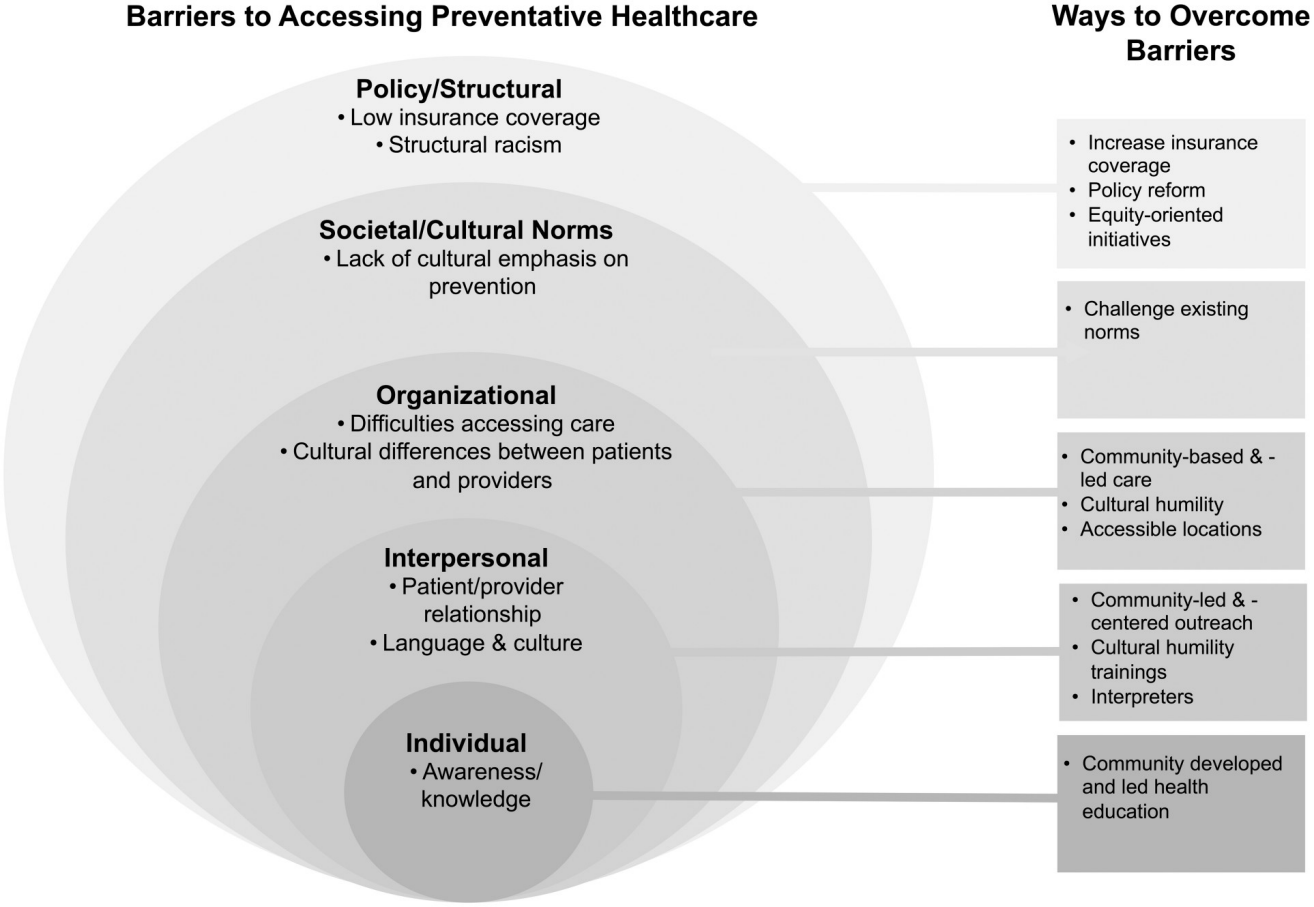
Socio-ecological model of barriers and potential solutions to accessing preventive health care among African-born individuals in King County.

Our analysis of the KIIs revealed key insights into the barriers and facilitators for access to preventive health care among African immigrant communities in King County (Fig 1 and Table 2); the findings are summarized below and illustrative indirect quotes for each identified theme are included in Table 2.

**Table 2:** Indirect quotes from key informant interviews describing barriers to accessing preventive health care, as well as the ways to help overcome them, among African immigrant communities in King County, WA (organized according to the domains in the socio-ecological model [37]).

Domain	Barriers to Accessing Preventive Health Care	Ways to Overcome Barriers
**Individual**	• Lack of individual awareness/knowledge of illnesses or available services 1. And education, talking with them, kind of like in school, we need to know when to do what. And most important, why? Why do we need to do mammography, this colon thing? *56-year-old female*, *Mexican*, *site manager* 2. More should be done on health education and access to information as many don’t even know what their rights and benefits are. *58-year-old male*, *Eritrean*, *medical professional*	• Health education 1. Educating the community and giving practical examples on how preventive medicine helps. Getting them involved in volunteering experiences and doing the basics so that way they feel they are making a difference and through that they feel compelled to use the preventive medicine. *24 years-old female*, *Kenyan*, *student* 2. Having more community-based health caregivers, involving culturally competent professionals to give us education about preventive health care. *54-year-old male*, *Ethiopian*, *taxi driver*
**Interpersonal**	• Differing language and cultural practices 1. The culture and language difference always make the care more difficult and the experience less enjoyable but it’s not because the care providers are bad. *32-year-old female*, *Somali*, *community organizer* • Suboptimal relationships between patients and providers 1. Not trusting the health care system; In hospitals and clinics my community’s ideas and opinions are not validated and this makes them less trusting of the health care system. *24-years-old female*, *Kenyan*, *student* • Discrimination based on various identities 1. I have heard and faced negative discriminatory treatment which I will say was based on where we are from, our competency, language barrier and race. *39-year-old female*, *Ethiopian*, *medical professional*	• More community-led and -centered health outreach 1. Involve community members, train people from the community who can give back. *58-year-old male*, *Eritrean*, *medical professional* • Cultural humility/competency trainings for providers 1. Health care providers should receive adequate training on the cultural and social value of the community it serves. *43-year-old female*, *Ethiopian*, *educator* • Greater availability of interpreters that speak native languages 1. Language barrier is a big problem- need to have more interpreters, providers who speak different languages. *49-year-old male*, *U*.*S*.*-born*, *youth educator*
**Organizational**	• Logistical and resource contraints to accessing care: Cost 1. The insurance system should be changed to make it more accessible and suitable for low income makers and immigrants. *41-year-old male*, *Ethiopian*, *airport attendant* Transportation 1. Accessible transportation, as far as many people do not know the city well. *37-year-old male*, *Ethiopian*, *medical professional* Time 1. Having services during the weekend helps because many people work during the week or normal clinic hours. *18-year-old female*, *Ethiopian*, *student* Lost wages due to time taken off from work 1. Our background makes us to give priority to work. *39-year-old female*, *Ethiopian*, *medical professional* • Lack of culturally-sensitive services or community-based/informed outreach efforts 1. [different] cultural and religious beliefs create miscommunication with care providers which finally may end up in lack of trust. *43-year-old female*, *Ethiopian*, *educator* • Mistrust of Western health systems and practices 1. Not trusting the health care system. Trusting herbal and traditional medicine more than standard health practices. *24-year-old female*, *Somali*, *student*	• Community-based and -led care 1. Churches and community centers would be the best places, but it takes a lot of time to get community leaders involved. *38-year-old female*, *Ethiopian*, *community health worker* 2. I don’t think there are any alternative health care providers. There are many non-practicing health care providers [in our communities] but they are not many practicing ones as the system tries to marginalize and reject them. Actually, that is one of the main problems and I believe has to be improved as it will increase trust [between community members and providers]. *58-year-old male*, *Eritrean*, *medical professional* 3. Many use the free clinic close to the apartment complex, which works well, because everyone from the community goes there, have built rapport and trust with the community. *18-year-old female*, *Ethiopian*, *student* • Cultural humility and culturally-competent care 1. It [health care system] should be more transparent and emphasis has to be given to culturally-competent care. *32-year-old female*, *Somali*, *medical professional* • Health services offered at or near where people live 1. I think people will tend to use it more because it avoids the transportation expenses and time obstacle. *24-year-old female*, *Somali*, *student* 2. People are much more likely to use services near their apartment. *33-year-old female*, *Ethiopian*, *homemaker* 3. If offered in the apartment building, makes people in the community feel like there people who care about them. *18-year-old female*, *Ethiopian*, *student*
**Societal/Cultural Norms**	• Lack of cultural emphasis on preventive health care 1. We come from a culture where we are more reactionary to health issues than proactive. *Female*, *West African*, *business owner*	• Challenge social/cultural norms in appropriate ways 1. Workshops on health insurance. Raise awareness on preventive health care benefits. *Female*, *West African*, *business owner* • Increase community involvement 1. First of all, acknowledge problem exists; do detailed research about it; involve community members, train people from the community who can give back, involve culturally competent professionals and of course involving donors for budgets is a problem. *58-*year*-old male*, *Eritrean*, *medical professional*
**Policy/structural**	• Difficulty obtaining insurance 1. Insurance, no knowledge on how to navigate the insurance system, the payment is unclear. Health care in the United States is often unaffordable and places a large financial burden on families. *26-year-old female Ethiopian*, *student* 2. The insurance system is not friendly to undocumented immigrants so that has to be changed. *43-year-old female*, *Ethiopian*, *educator*	• Expand insurance access 1. The insurance system is one of the things that can be changed but I think it is a large scale problem. *26-year-old female*, *Ethiopian*, *student* 2. I also believe there should be universal health care for everyone. *37-year old female*, *Ethiopian*, *medical professional*

#### Individual domain: Lack of knowledge and awareness requires increased health education efforts

Within the individual domain, participants consistently identified lack of individual awareness or knowledge about illnesses and the U.S. health system as key barriers. An apartment complex manager involved in community health outreach for African immigrants mentioned:

*And education, talking with them, kind of like in school, we need to know when to do what. And most important, why? Why do we need to do mammography, this colon thing?*

*(56-year-old female, Mexican, site manager)*

Participants specifically mentioned that their communities had limited awareness of mental illnesses, especially as represented within their own cultural contexts, as well as the need for preventive health screening for chronic conditions. To that effect, they identified health education, especially when led by culturally compentent professionals from their own communities, as a major way of overcoming these barriers. One event participant suggested:

*Educating the community and giving practical examples on how preventive medicine helps. Getting them involved in volunteering experiences and doing the basics so that way they feel they are making a difference and through that they feel compelled to use the preventive medicine*.

*(24 years-old female, Kenyan, student)*

#### Interpersonal domain: Trusting relationships with health care providers are hindered by language, cultural differences, and discrimination

In the interpersonal domain, participants reported that differing language and cultural practices often complicated relationships between patients and providers. One participant specifically mentioned both witnessing and facing overt discrimination based on race, language, and culture:

*I have heard and faced negative discriminatory treatment which I will say was based on where we are from, our competency, language barrier and race*.

*(39-year-old female, Ethiopian, medical professional)*

A common suggestion for overcoming these barriers was placing a central focus on community-led and -centered health outreach. Participants also proposed improving access to interpreters and providing training for health care providers about working with African immigrant populations.

#### Organizational domain: Perceived cost and time spent accessing care may inhibit uptake of health services

Within the organizational domain, participants frequently mentioned logistical or resource barriers to accessing services (e.g. cost, time, transportation, or work conflicts), though others included longstanding mistrust of Western health systems and practices. Ways to overcome these set of barriers were similar as in the interpersonal domain but also focused on bringing health services closer to where people live or work. One health fair participant identified a nearby community clinic as a popular choice:

*Many use the free clinic close to the apartment complex, which works well, because everyone from the community goes there, have built rapport and trust with the community*.

*(18-year-old female, Ethiopian, student)*

#### Societal/cultural domain: Low cultural emphasis on preventive health reduces engagement in health promotion

Low cultural prioritization of preventive health care was identified as societal/cultural barrier, which could be overcome by challenging these norms in culturally-appropriate ways and increasing community involvement in health promotion activities. A medical professional participant said:

*First of all, acknowledge problem exists; do detailed research about it; involve community members, train people from the community who can give back, involve culturally competent professionals and of course involving donors for budgets is a problem*.

*(58-year-old male, Eritrean, medical professional)*

#### Policy/structural domain: Removing barriers to health insurance coverage could improve engagement in care

Lastly, within the policy and structural domain, lack of health insurance was a frequently identified barrier. Participants noted that, without insurance, health care costs in the U.S. are prohibitively high. A health fair participant said:

*Insurance, no knowledge on how to navigate the insurance system, the payment is unclear. Health care in the United States is often unaffordable and places a large financial burden on families*.

*26-year-old female, Ethiopian, student*

Structural racism also emerged as a contributing factor for African-born individuals’ hesitancy in engaging with the healthcare systems in the U.S. Several perceived that engaging with and navigating systems to obtain insurance coverage was unfriendly to recent immigrants, especially if undocumented. Several participants mentioned that policies providing universal health coverage would remove barriers to engagement in care.

#### Participants differed on perceived gender roles in health care coordination

To better understand any gendered norms around health care seeking behaviors, we probed a few gender-based themes related to accessing health care. When asked which family member was generally responsible for coordinating health care needs for the family, participants’ responses varied. Some participants identified that male family members (e.g. fathers) were more likely to coordinate the family’s health care needs because of existing patriarchal structures within the community, men having better English proficiency, and more awareness and information about the health care system. For example, when asked who in the family coordinated health care needs, one community leader responded:

*The father mostly. But responsibility is shared equally if both parents are well educated enough to navigate the health system. Since most fathers work their English proficiency tends to be higher, and this makes engaging with the health care systems easier for them*

*(38-year-old female, Ethiopian, community health worker)*.

Other participants identified women or mothers to be the ones who coordinate health care needs, as women are more involved in childcare and perceived as better at multitasking and planning. On the other hand, some participants believed that the role of family health care coordinator depended on the level of education, language skills, personality of the family member, and awareness of the health care system. Most participants felt women were also more likely to engage in preventive health care due to reproductive health care needs, such as during a pregnancy. Participants were also asked about which age group was more likely to see a doctor or dentist regularly, and most participants felt children and the elderly were more likely than adults to seek such care regularly, but this did not vary by gender.

## Discussion

Our analysis identified several barriers to accessing preventive health care among African immigrant communities in King County, WA. Key insights from our analysis include the need for community-centered and -led care that is culturally appropriate in order to navigate and overcome the barriers to preventive health care identified. This theme emerged repeatedly as a potential solution for the barriers identified in several domains. For example, health education efforts should be tailored to the specific learning styles of the engaged communities so that their health literacy can be optimized. Large scale structural reforms were also suggested in response to barriers relating to insurance and cost of care, though participants were less concrete on how to address other issues such as structural racism. Additionally, changes in sociocultural attitudes were often proposed as necessary to counteract stigma around mental health, HIV/AIDS, immigration status, and discrimination faced in the health care system. Finally, while some participants identified gendered norms regarding roles and responsibilities for health care navigation, others remarked that educational attainment and English proficiency, regardless of gender, were more important for coordinating family health needs.

The emphasis on community-centered care suggests a potential strategy for improving engagement in preventive health services. The community-hosted health events at which our study took place represent an important effort to meet medical needs outside the formal health care system [38]. As one participant noted, however, these efforts need reliable funding support to provide effective services. Health systems should interface with faith-based institutions and nonprofit organizations that routinely provide services to these communities in order to build effective and long-lasting relationships. Community venues provide opportunities for both health promotion activities and early linkage to primary care; in addition, home-based care models may facilitate innovative models for delivery of health care. While home visits have been adopted for refugee groups, home-based primary care remains underexplored in the U.S. for African immigrant communities as a whole [39]. Recruitment of African-born community health workers to provide linguistic and culturally appropriate care can help overcome current barriers related to health care navigation and improve retention in care [40]. In addition, expanding access to interpreter services, streamlining pathways for international medical graduates to practice in the U.S., and emphasizing cultural humility in medical education programs may improve the ability of the health care system to provide effective care for African-born individuals.

Difficulties obtaining or navigating insurance were frequently reported as structural barriers to accessing preventive health. Individuals in the U.S. without health insurance generally must pay for preventive services out-of-pocket. Free or subsidized services can sometimes be obtained via a patchwork of federally qualified community health centers, non-profit or faith-based clinics, or temporary free clinics. Nevertheless, uninsured individuals are substantially less likely to receive preventive services [41], suggesting that increasing insurance coverage among immigrant populations may improve chronic disease health outcomes. Our results are consistent with a prior study of African immigrants attending a health fair that found cost of care as a common barrier among a population with low levels of health insurance [10]. Barriers to insurance access include restrictive eligibility critiera, such as a five-year waiting period for Medicaid coverage, and the implementation of a redefined public charge rule is anticipated to have “chilling effects” on access to public assistance benefits, including health insurance [42–46]. Policies that impede immigrant use of welfare support or other public services have been linked to worse health outcomes [47, 48]. These policies may also reinforce cultural attitudes that emphasize self-reliance and discourage use of public preventive care services.

Our results have several potential limitations. Most of the community events we were invited to were hosted by the local Ethiopian or Somali communities, and the majority of participants in the KIIs were born in Ethiopia. Our results may therefore not necessarily generalize to African communities from other countries. Additionally, the KIIs were conducted among a convenience sample within King County, WA and may not capture nuances specific to communities located in other cities or countries. Our snowball sampling approach likely resulted in respondents with more education and familiarity with the health care system and therefore may not have been representative of all African-born individuals in the study area. We relied on interviewer field notes instead of recordings, which could have resulted in memory errors or unconscious bias based on interviewer interpretation. While we choose the socio-ecological model to organize results once themes emerged, we did not specifically design our study around that model, and as a result our work is neither theory-driven nor validating an existing theory. Nevertheless, the consistency of barriers reported across participants suggests that our approach identified common experiences for this population.

## Conclusions

In summary, despite a rising burden of NCDs in African countries and among African immigrants, African-born individuals encounter multilevel barriers to accessing preventive health care. Further reseach into new community-led service delivery approaches is warranted to improve access to care and health outcomes among African immigrant communities in the U.S.

## Supporting information

S1 File (PDF)

S2 File (PDF)
