# Supplementary material for: Barriers to accessing preventive health care among African-born individuals in King County, Washington: A qualitative study involving key informants
Source: PLoS One. 2021 May 10;16(5):e0250800. doi: 10.1371/journal.pone.0250800 (PMC8109781; doi:10.1371/journal.pone.0250800)
Supplement: S2 File — (PDF) [file pone.0250800.s002.pdf]

Key Informant Interview Guide Today's date: _________Interviewer: ________________

A. Background information

Age: _____________________________ Gender (circle one): Female Male Other

Country of birth: ____________________ Primary language: _________________________________

Occupation: _______________________ Health board/organization affiliation: _________________

B. General healthcare

Question/topic area	Responses/notes
1) What are the biggest barriers for African immigrants in engaging in preventative healthcare in the Seattle area?<br>- How improve?<br>- Are there any ways around these barriers that people in your community/culture have used? How successful was this?	
2) What aspects of the healthcare system could be changed or improved to help African immigrants be better engaged in preventative healthcare?<br>- What have you heard or noticed how members of your community/culture are generally treated in formal healthcare settings (clinics, hospital, etc.?)	
3) In your community/culture, which member of the family member tends to coordinate healthcare needs for the family?<br>- What works well about this structure? What is difficult?<br>- Are children or adults more likely to see the doctor and/or dentist more regularly?	
4) Do you feel that men or women are more likely to seek preventative healthcare in your community/culture?<br>- What works well about this structure? What is difficult?<br>- How do women find care during pregnancy and childbirth? What have you experienced or heard about women's experiences with healthcare providers during pregnancy and childbirth? How do you feel this experience would be different if a woman was known to be HIV positive?	
5) Are there any alternative healthcare providers that are popular in your community/culture?<br>- Who has access to these providers, and how?<br>- How do people go about paying these providers?<br>- What kinds of services do they provide?<br>- Are there many practicing or non-practicing mainstream healthcare providers in your community who provide care for other community members?	
6) What types of locations are convenient for your community members to access healthcare services? How likely are people in your community/culture to use healthcare services offered in or near their apartment building?	
